# Specialization of the *Drosophila* nuclear export family protein Nxf3 for piRNA precursor export

**DOI:** 10.1101/gad.328690.119

**Published:** 2019-09-01

**Authors:** Emma Kneuss, Marzia Munafò, Evelyn L. Eastwood, Undine-Sophie Deumer, Jonathan B. Preall, Gregory J. Hannon, Benjamin Czech

**Affiliations:** 1 Cancer Research UK Cambridge Institute, Li Ka Shing Centre, University of Cambridge, Cambridge CB2 0RE, United Kingdom;; 2 Cold Spring Harbor Laboratory, Cold Spring Harbor, New York 11724, USA

**Keywords:** transposon control, PIWI proteins, piRNA clusters, nuclear export factor, RNA export

## Abstract

The PIWI-interacting RNA (piRNA) pathway is a conserved small RNA-based immune system that protects animal germ cell genomes from the harmful effects of transposon mobilization. In *Drosophila* ovaries, most piRNAs originate from dual-strand clusters, which generate piRNAs from both genomic strands. Dual-strand clusters use noncanonical transcription mechanisms. Although transcribed by RNA polymerase II, cluster transcripts lack splicing signatures and poly(A) tails. mRNA processing is important for general mRNA export mediated by nuclear export factor 1 (Nxf1). Although UAP56, a component of the transcription and export complex, has been implicated in piRNA precursor export, it remains unknown how dual-strand cluster transcripts are specifically targeted for piRNA biogenesis by export from the nucleus to cytoplasmic processing centers. Here we report that dual-strand cluster transcript export requires CG13741/Bootlegger and the *Drosophila* nuclear export factor family protein Nxf3. Bootlegger is specifically recruited to piRNA clusters and in turn brings Nxf3. We found that Nxf3 specifically binds to piRNA precursors and is essential for their export to piRNA biogenesis sites, a process that is critical for germline transposon silencing. Our data shed light on how dual-strand clusters compensate for a lack of canonical features of mature mRNAs to be specifically exported via Nxf3, ensuring proper piRNA production.

## Abstract (precis)

Here, Kneuss et al. investigated how dual-strand cluster transcripts are targeted for piRNA biogenesis by export from the nucleus to cytoplasmic processing centers. The authors identify Bootlegger and Nxf3 as novel components of the piRNA precursor processing pathway required for transposon silencing in the fly germline.

Transposable elements represent a major threat to genome integrity. In the animal germline, transposon mobilization is prevented by a conserved small RNA-based immune system, the PIWI-interacting RNA (piRNA) pathway. piRNAs are 23- to 30-nucleotide (nt) small RNAs that associate with Argonaute proteins of the PIWI clade (Czech et al. 2018; Ozata et al. 2019). The majority of piRNAs are produced from discrete genomic loci, called piRNA clusters, which are composed of transposon remnants and thus constitute a genetic memory of past transposon exposure (Brennecke et al. 2007). In *Drosophila*, piRNA clusters give rise to piRNA precursor transcripts from either one (unistrand clusters) or both (dual-strand clusters) genomic strands (Malone et al. 2009; Mohn et al. 2014). The processing of precursor transcripts into mature piRNAs is a cytoplasmic event occurring in nuage, cytoplasmic structures adjacent to the nucleus that are enriched for piRNA biogenesis components (Lim and Kai 2007; Malone et al. 2009). Therefore, precursor RNAs require export from the nucleus for conversion into small RNAs. However, how precursors are identified for export and selectively trafficked to nuage remains a mystery.

Canonical mRNA export in eukaryotes relies on nuclear export factor 1 (Nxf1) and its cofactor, Nxt1 (NTF2-related export protein 1) (Herold et al. 2001; Köhler and Hurt 2007; Stewart 2010). However, mRNA export is a highly regulated process, occurring only on transcripts that have undergone mRNA processing. This includes the formation of the 5′ cap, splicing, and 3′ end processing, which involves endonucleolytic cleavage followed by polyadenylation (Le Hir et al. 2001; Cheng et al. 2006; Köhler and Hurt 2007; Yoh et al. 2007). Completion of mRNA processing leads to the recruitment of the transcription and export complex (TREX), which is a prerequisite for association with the Nxf1–Nxt1 heterodimer. Unistrand clusters, such as *flamenco* (*flam*), are capped, spliced, and polyadenylated and consequently follow canonical export rules, relying on Nxf1–Nxt1 (Goriaux et al. 2014; Mohn et al. 2014; Dennis et al. 2016).

In contrast, germline-specific dual-strand clusters produce noncanonical transcripts that, while capped and transcribed by RNA polymerase II (Pol II), lack splicing signatures and poly(A) tails (Mohn et al. 2014; Zhang et al. 2014; Andersen et al. 2017). Furthermore, H3K4me2 patterns typical of active promoters are missing on dual-strand clusters. Instead, these loci are embedded within heterochromatin and feature H3K9me3 marks, which are normally not correlated with transcription (Mohn et al. 2014; Zhang et al. 2014). Recent work has uncovered the machinery that facilitates heterochromatin-dependent dual-strand cluster transcription (Mohn et al. 2014; Zhang et al. 2014; Andersen et al. 2017). The heterochromatin protein 1a (HP1a) homolog Rhino (Rhi) specifically associates with dual-strand clusters through its H3K9me3-binding domain (Klattenhoff et al. 2009; Mohn et al. 2014; Zhang et al. 2014). Rhi interacts with the linker protein Deadlock (Del), which was shown to recruit Moonshiner (Moon), a homolog of the transcription initiation factor II A (TFIIA) (Andersen et al. 2017). Moon promotes transcription initiation of dual-strand cluster loci via its interaction with the TATA-binding protein (TBP)-related factor TRF2. Del also interacts with Cutoff (Cuff), a protein with similarity to the Rai1 transcription termination factor (Pane et al. 2011; Mohn et al. 2014; Zhang et al. 2014; Chen et al. 2016). Cuff suppresses splicing and Pol II termination and is thought to protect dual-strand piRNA cluster transcripts from degradation by the exonuclease Rat1 (Zhang et al. 2014; Chen et al. 2016). Thus, this Rhi-anchored complex generates transcripts that lack signatures of canonical mRNAs. UAP56, a DEAD-box helicase also involved in canonical mRNA splicing and export, was shown to associate with piRNA precursor transcripts (Zhang et al. 2012, 2018). Colocalization of UAP56 with the sites of dual-strand cluster transcription and a close association with the perinuclear DEAD-box helicase Vasa (Vas) suggested that this protein is involved in coupling precursor synthesis to downstream processing (Zhang et al. 2012).

The noncanonical transcription of dual-strand clusters (absence of splicing and polyadenylation) suggests that their RNA products require an alternative export machinery that does not involve Nxf1/Nxt1. Here, we show that piRNA cluster export depends on a germline-specific paralog of Nxf1, Nxf3, that is recruited to cluster transcripts through its interaction with CG13741/Bootlegger and the Rhi–Del–Cuff (RDC) complex. Both CG13741/Bootlegger and Nxf3 are essential for piRNA-guided transposon repression in germ cells. Nxf3-dependent export of piRNA precursors from the nucleus to nuage likely involves Nxt1 and uses a Crm1 (chromosomal maintenance 1)-dependent mechanism. Together, our data shed light on how noncanonical piRNA precursor transcripts are exported from their heterochromatic source loci to the processing sites in the cytoplasm and how specific components of the nuclear export machinery are adapted to facilitate this.

## Results

### CG13741 and Nxf3 are germline-specific piRNA factors involved in dual-strand cluster biology

**Figure 1.**
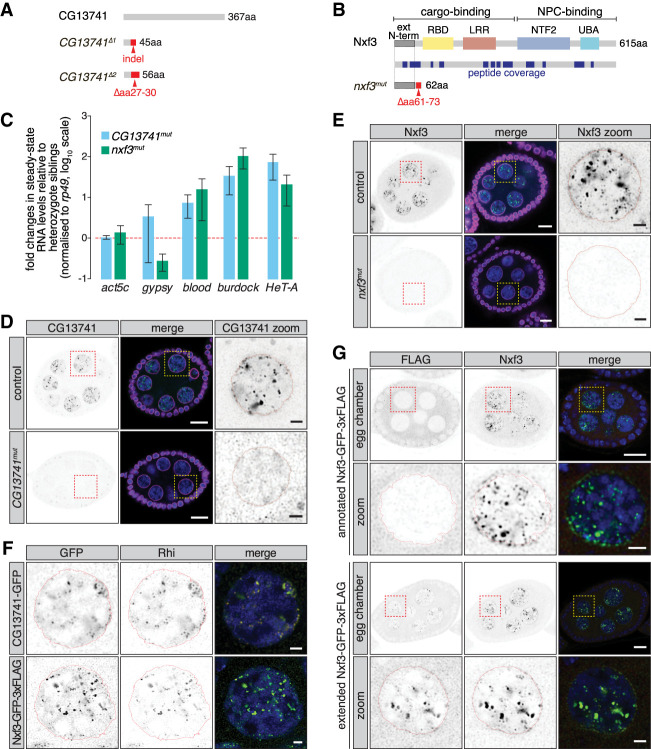
CG13741 and Nxf3 impact dual-strand piRNA cluster biology. (*A*) Schematic representation of CG13741 as well as *CG13741^Δ1^* and *CG13741^Δ2^* mutants. (*B*) Cartoon displaying the Nxf3 domain structure and the generated *nxf3*^*mut*^ allele. Peptide coverage obtained by Nxf3 pull-down followed by mass spectrometry is indicated in blue. (*C*) Bar graphs showing fold changes in steady-state RNA levels of a housekeeping gene (*act5c*) and soma-specific (*gypsy*), intermediate (*blood*), and germline-specific (*HeT-A* and *burdock*) transposons in total ovarian RNA from the indicated genotypes (relative to heterozygote siblings and normalized to *rp49*). Error bars indicate standard deviation. *n* = 3. (*D*) Expression and localization of CG13741 in an egg chamber of control and *CG13741*^*mut*^ mutant flies are shown by immunofluorescence. A zoomed-in view of the indicated nurse cell nucleus is shown at the *right*. (Green) CG13741; (magenta) Lamin; (blue) DNA. Scale bar for egg chamber, 10 µm; scale bar for zoom-in, 2 µm. (*E*) As in *D* but showing the expression and localization of Nxf3 in control and *nxf3*^*mut*^ egg chambers. (Green) Nxf3; (magenta) Lamin; (blue) DNA. Scale bar for egg chamber, 10 µm; scale bar for zoom-in, 2 µm. (*F*) As in *D* but showing expression and localization of GFP-tagged CG13741 and GFP-tagged Nxf3 costained with Rhi. (Green) GFP; (red) Rhi; (blue) DNA. Scale bar, 2 µm. (*G*) As in *D* but showing expression and localization of the annotated and extended Nxf3 sequence tagged C-terminally with GFP-3xFlag and costained with an antibody to endogenous Nxf3. (Green) Nxf3; (red) Flag; (blue) DNA. Scale bar for egg chamber, 10 µm; scale bar for zoom-in, 2 µm.

Genome-wide screens in *Drosophila* have revealed the genetic framework of the piRNA pathway in somatic and germ cells (Czech et al. 2013; Handler et al. 2013; Muerdter et al. 2013). Since proteins involved in dual-strand piRNA cluster biology will specifically compromise transposon silencing in germ cells, we focused on two uncharacterized germline-specific screen hits: CG13741 and Nxf3 (Czech et al. 2013). CG13741 is a 42-kDa protein without identifiable domains (Fig. 1A) that is expressed predominantly in ovaries (Supplemental Fig. S1A). Nxf3 is also expressed preferentially in ovaries (Supplemental Fig. S1A) and is a member of the nuclear export factor family, which in *Drosophila* includes three additional proteins (Supplemental Fig. S1B). Ubiquitously expressed Nxf1 is responsible for bulk mRNA export (Herold et al. 2001), while ovary-enriched Nxf2 functions in piRNA-guided transcriptional transposon silencing (Batki et al. 2019; Fabry et al. 2019; Murano et al. 2019; Zhao et al. 2019). Nxf3 and the testis-specific Nxf4 have not yet been ascribed functions. Similar to other members of this protein family, Nxf3 contains an RNA-binding domain (RBD), leucine-rich repeats (LRRs), a NTF2-like domain (NTF2), and a diverged ubiquitin-associated domain (UBA) (Fig. 1B). While Nxf1 and Nxf2 were identified to be required for transposon silencing in somatic and germline cells, Nxf3 was required only in germ cells (Czech et al. 2013; Handler et al. 2013; Muerdter et al. 2013).

Using CRISPR/Cas9, we generated *CG13741* and *nxf3* mutant flies. We obtained two *CG13741* mutant alleles, *CG13741^Δ1^* and *CG13741^Δ2^*, which harbor premature stop codons that disrupt the CG13741 ORF from amino acid 27 onward (Fig. 1A). For better readability, we refer to *CG13741* mutant and *trans*-heterozygote alleles as *CG13741*^*mut*^. For Nxf3, we recovered one mutant allele, *nxf3*^*mut*^, that leads to a premature stop codon that disrupts the ORF just downstream from the annotated start codon (Fig. 1B). Western blots on ovarian lysates from control, heterozygous, and *nxf3* homozygous mutant flies confirmed this allele as null (see below; Supplemental Fig. S1C). *CG13741* and *nxf3* mutants were female sterile, with homozygous mutant females laying slightly fewer eggs (∼70% of control females for both mutants), of which few (1.9% for *CG13741*^*mut*^) or none (0.0% for *nxf3*^*mut*^) hatched (Supplemental Fig. S1D). Flies mutant for either *CG13741* or *nxf3* were compromised in the repression of intermediate (*blood*) and germline-specific (*burdock* and *HeT-A*) transposons, while soma-specific transposable elements (*gypsy*) remained largely unchanged (Fig. 1C). We did not detect changes in the localization of piRNA-binding Argonaute proteins Piwi, Aub, or Ago3 in *CG13741*^*mut*^ and *nxf3*^*mut*^ ovaries (Supplemental Fig. S1E). Thus, our data suggest that CG13741 and Nxf3 are important for transposon repression and could function in germline piRNA cluster biology.

In order to investigate their function in transposon control, we examined the expression pattern and subcellular localization of CG13741 and Nxf3 using polyclonal antibodies that we generated. Both CG13741 and Nxf3 localized to discrete foci in nurse cell nuclei (Fig. 1D,E), which is highly reminiscent of staining patterns observed for RDC complex subunits (Klattenhoff et al. 2009; Pane et al. 2011; Mohn et al. 2014), while follicle cells lacked both proteins. Both CG13741 and Nxf3 colocalize with nuclear foci stained by Rhi (Fig. 1F), supporting the hypothesis that they are involved in dual-strand piRNA cluster biology. Unlike RDC complex components, a fraction of Nxf3 (and, to a lesser extent, CG13741) also localizes to perinuclear foci that form ring-like structures outside nuclei and strongly resemble nuage (Fig. 1D,E), suggesting a possible role in piRNA precursor export.

To facilitate further biochemical studies, we created a Nxf3 cDNA transgene with a C-terminal epitope tag (GFP-3xFlag). In contrast to the staining observed for the endogenous protein, the Nxf3 transgene was aberrantly localized to the cytoplasm (Fig. 1G), prompting us to examine the Nxf3 genomic locus. We noticed the presence of an additional 56 in-frame amino acids with an initiator methionine at the N terminus. Immunoprecipitation of Nxf3 with our antibody followed by mass spectrometry revealed peptides specific to and overlapping with the putative N-terminal extension (Fig. 1B). We generated flies expressing the N-terminally extended Nxf3 sequence tagged C-terminally with GFP-3xFlag and found that the extended version colocalized with endogenous Nxf3 in germ cells, as shown by immunofluorescence staining (Fig. 1G). We therefore conclude that the extended form of Nxf3 likely represents the functional and full-length protein. Unless specified otherwise, we used the extended version of Nxf3 for subsequent experiments and refer to this fusion as Nxf3-GFP-3xFlag.

### CG13741 and Nxf3 recruitment to dual-strand clusters depends on the RDC complex

**Figure 2.**
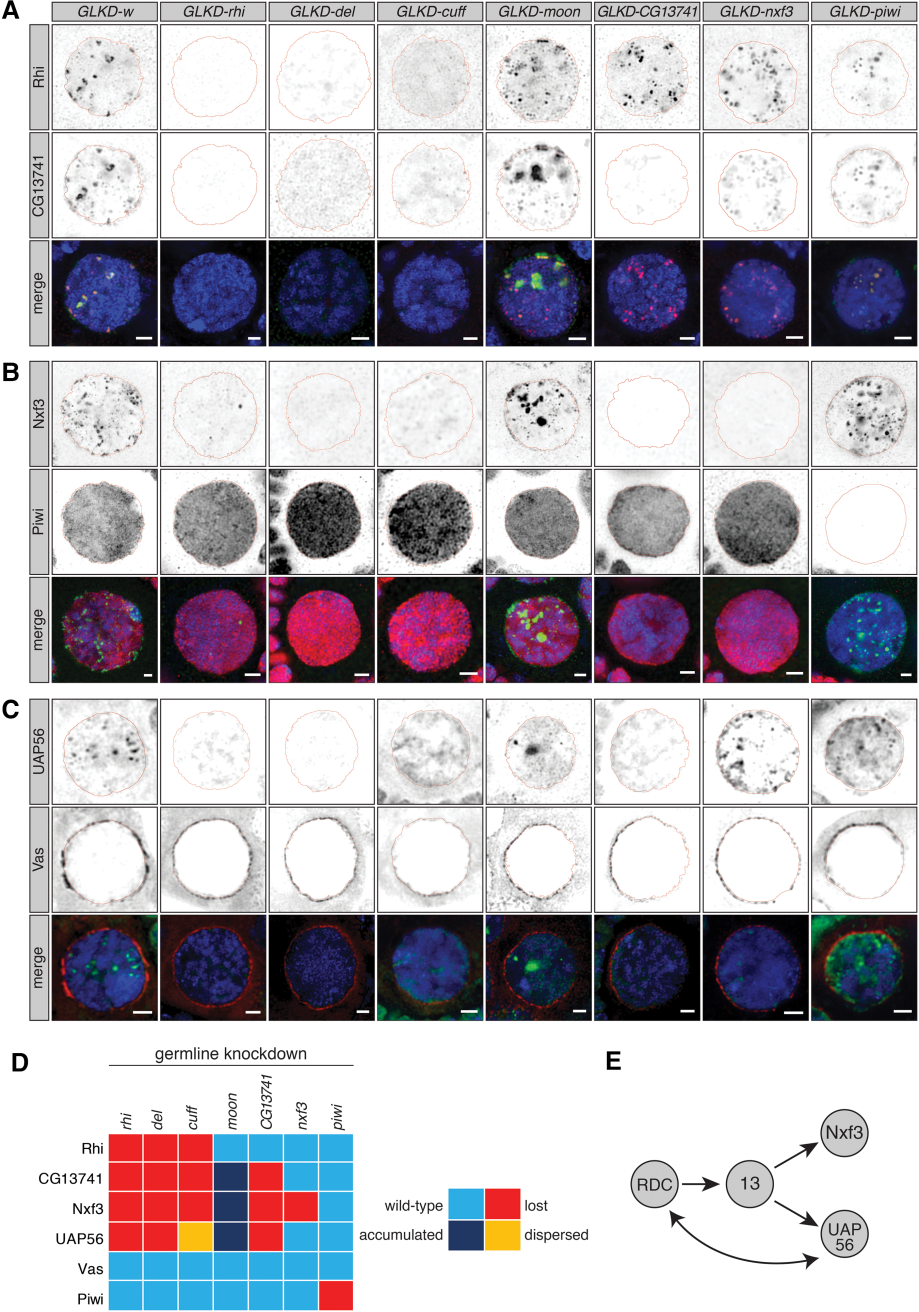
Hierarchy of dual-strand piRNA cluster factors. (*A*) Expression and localization of Rhi and CG13741 in nurse cell nuclei upon germline-specific knockdown (GLKD) of the indicated factors are shown by immunofluorescence. (Green) CG13741; (red) Rhi; (blue) DNA. Scale bar, 2 µm. (*B*) As in *A* but showing the expression and localization of Piwi and Nxf3. (Green) Nxf3; (red) Piwi; (blue) DNA. Scale bar, 2 µm. (*C*) As in *A* but showing the expression and localization of UAP56 and Vas. (Green) UAP56; (red) Vas; (blue) DNA. Scale bar, 2 µm. (*D*) Heat map summarizing the data shown in *A*–*C*. (*E*) Cartoon showing the hierarchy of RDC, CG13741, UAP56, and Nxf3.

The RDC complex has an essential role in dual-strand cluster expression, as it licenses clusters and recruits the transcription machinery to these loci (Mohn et al. 2014; Zhang et al. 2014; Andersen et al. 2017). To understand the functional hierarchy of CG13741, Nxf3, and other piRNA pathway components, we depleted factors implicated in piRNA cluster biology in germ cells and examined the localization of CG13741, Nxf3, and other piRNA pathway components by immunofluorescence. Germline knockdown of all factors led to robust derepression of intermediate and germline-specific transposons, indicating robust depletion (Supplemental Fig. S2A). We first analyzed the dependency of Rhi and CG13741 on other known piRNA factors affecting dual-strand clusters by depleting these specifically in the germ cells of the ovary (Fig. 2A). Nuclear foci stained by Rhi were lost in germline knockdowns of components of the RDC complex, as reported previously (Mohn et al. 2014; Zhang et al. 2014). Depletion of Moon had no effect on Rhi recruitment, consistent with its function downstream from RDC (Andersen et al. 2017). Similarly, germline knockdown of *CG13741* and *nxf3* had no effect on Rhi localization, suggesting that both factors function downstream from the RDC complex (Fig. 2A).

In control knockdowns, CG13741 was detected in nuclear foci that overlap with Rhi, and a weak signal was detected outside nuclei (Fig. 2A). Recruitment of CG13741 to nuclear foci was dependent on all RDC complex components and completely lost upon germline-specific depletion of CG13741 itself (Fig. 2A). The localization of CG13741 to nuclear foci was unaffected by depletion of Nxf3. Interestingly, CG13741 signal outside of the nucleus was lost upon *nxf3* knockdown, suggesting a role of Nxf3 in the export of CG13741 out of the nucleus (Fig. 2A).

In control knockdowns, Nxf3 localized to discrete foci in nurse cell nuclei, likely piRNA cluster loci, as well as to nuage (Fig. 2B). Upon depletion of RDC complex components, all discrete concentrations of Nxf3 signal were lost. Additionally, the localization of Nxf3 to nuclear foci was dependent on the presence of CG13741 (Fig. 2B). Earlier work reported that Rhi depletion leads to loss of UAP56 at piRNA cluster loci (Zhang et al. 2012). Thus, we probed a potential role of CG13741 and Nxf3 in the recruitment of UAP56 (Fig. 2C). As expected, UAP56 recruitment depended on all components of the RDC complex and also on CG13741. In contrast, germline knockdowns of *nxf3* had no effect on the localization of UAP56, suggesting that CG13741 acts upstream of both Nxf3 and UAP56 (Fig. 2C).

Of note, germline knockdown of *moon* led to pronounced accumulation of CG13741, Nxf3, and UAP56 in nuclear foci that partially costained for Rhi (Fig. 2A–C). At present, the nature of such loci is unclear.

Depletion of piRNA dual-strand cluster factors did not eliminate nuclear Piwi signal (Fig. 2B), and depletion of Piwi from ovarian germ cells did not impact on factors involved in dual-strand piRNA cluster transcription, as reported (Akkouche et al. 2017). Conversely, knockdown of *piwi* did not alter the localization of CG13741, UAP56, or Nxf3, suggesting that Piwi is not required, at least in the short term, for formation of the signals that recruit these proteins to cluster loci (Fig. 2A–C). Last, Vas localization was not affected by loss of any of the factors studied, consistent with its role in assembling nuage and a potential function downstream from piRNA cluster transcription and export (Fig. 2C).

The role of UAP56 in Nxf3 and CG13741 recruitment was also tested using *uap56^sz15′/28^* mutants. Only UAP56 localization at clusters is affected in these mutants. Nxf3 and CG13741 levels were strongly reduced in the mutants compared with controls, while weak signals were still detected at discrete nuclear foci (Supplemental Fig. S2B). Furthermore, CG13741 and Rhi colocalization was maintained in *uap56^sz15′/28^* mutants (Supplemental Fig. S2B, left).

Considered together, our data indicate that CG13741 and Nxf3 recruitment is dependent on the presence of the RDC complex. CG13741 is essential for the correct localization of both Nxf3 and UAP56 to dual-strand piRNA clusters (Fig. 2D,E), whereas Nxf3 in turn could promote translocation of some CG13741 protein outside of the nucleus. Given the involvement of CG13741 in the biology of dual-strand piRNA clusters, we named it, by mutual agreement, Bootlegger (ElMaghraby et al. 2019).

### Bootlegger and Nxf3 are required for piRNA production from dual-strand clusters

**Figure 3.**
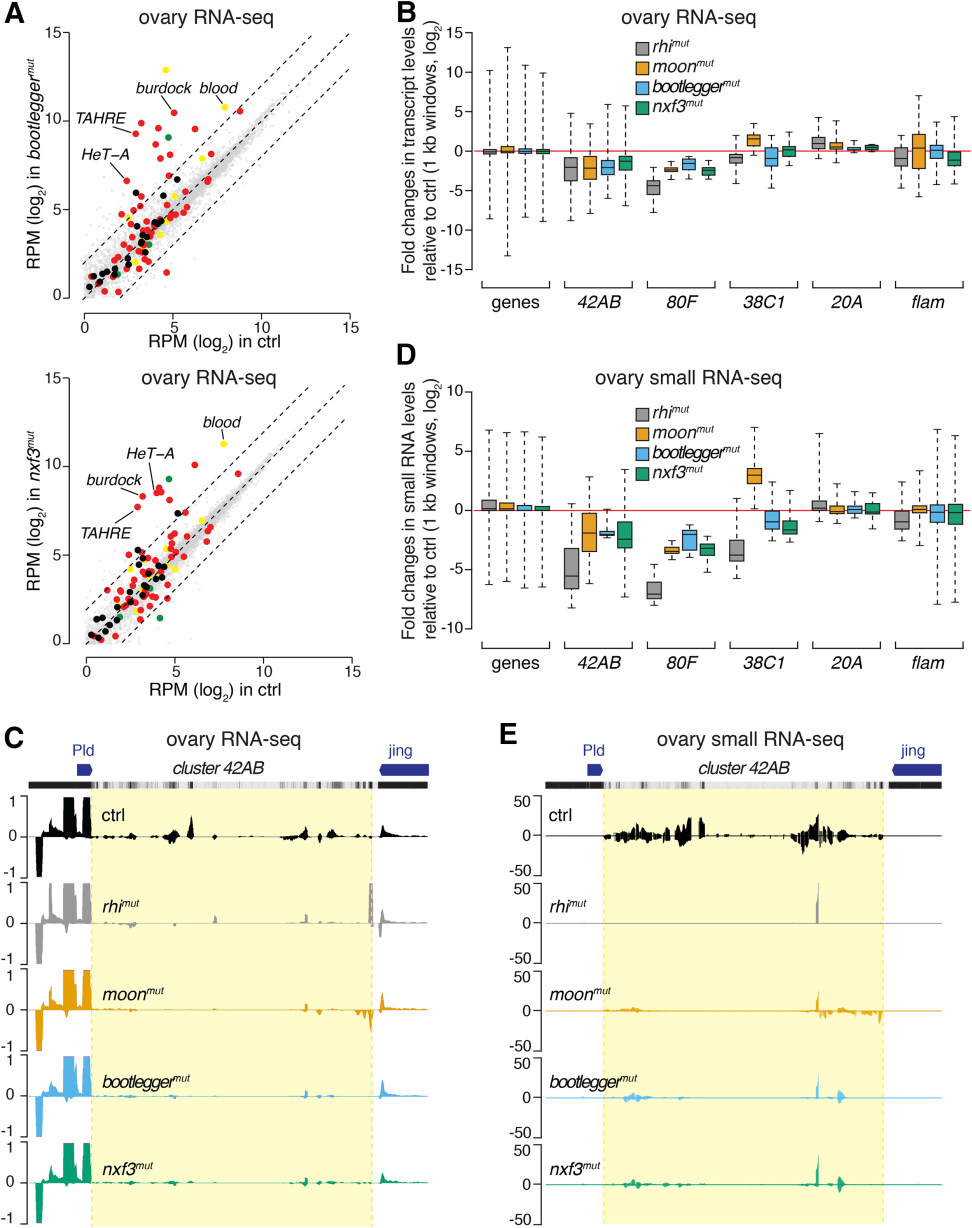
Bootlegger and Nxf3 are essential for piRNA production. (*A*) Scatter plots showing expression levels (reads per million sequenced reads [RPM]) of genes (in gray) and transposons (germline-specific in red, intermediate in yellow, soma-specific in green, and other transposons in black) from total RNA from ovaries of the indicated genotypes (*bootlegger*^*mut*^ [*top*] and *nxf3*^*mut*^ [*bottom*]; *n* = 3) compared with control flies. Dashed lines indicate fourfold expression changes. (*B*) Box plots showing changes in RNA levels between the indicated genotypes and control in ovarian RNA-seq for the indicated categories (1-kb bins for piRNA cluster transcripts *42AB*, *80F*, *38C1*, *20A*, and *flam*). (Gray) *rhi*^*mut*^; (orange) *moon*^*mut*^; (blue) *bootlegger*^*mut*^; (green) *nxf3*^*mut*^. (*C*) University of California at Santa Cruz (UCSC) genome browser shot displaying profiles of RNA-seq levels of reads uniquely mapping to cluster *42AB* and flanking euchromatic regions in the indicated genotypes. (Black) control; (gray) *rhi*^*mut*^; (orange) *moon*^*mut*^; (blue) *bootlegger*^*mut*^; (green) *nxf3*^*mut*^. The mappability of 50-bp reads is shown *above*. (*D*) As in *B*, but shown are box plots of small RNA-seq for the indicated categories (1-kb bins for piRNA cluster transcripts *42AB*, *80F*, *38C1*, *20A*, and *flam*). (Gray) *rhi*^*mut*^; (orange) *moon*^*mut*^; (blue) *bootlegger*^*mut*^; (green) *nxf3*^*mut*^. (*E*) As in *C*, but shown are piRNAs (23- to 29-nt in size) uniquely mapping to cluster *42AB* and flanking euchromatic regions in the indicated genotypes. (Black) Control; (gray) *rhi*^*mut*^; (orange) *moon*^*mut*^; (blue) *bootlegger*^*mut*^; (green) *nxf3*^*mut*^. The mappability of 25-bp reads is shown *above*.

To explore the genome-wide impact of *bootlegger* and *nxf3* mutations, we performed RNA sequencing (RNA-seq) from ovaries of mutant flies and compared these with control flies. The expression of protein-coding genes was only mildly affected in *bootlegger*^*mut*^ (*r*^2^ = 0.96) or *nxf3*^*mut*^ (*r*^2^ = 0.99), while loss of Bootlegger or Nxf3 led to a strong derepression of germline (*burdock*, *HeT-A*, and *TAHRE*) and intermediate (*blood*) transposons (Fig. 3A). Somatic transposons were not changed in *bootlegger* and *nxf3* mutants, as expected based on their expression patterns (Fig. 3A). Fourteen out of 60 germline transposon families (above the expression threshold) showed more than fourfold up-regulation in *bootlegger* mutants (Fig. 3A, top). In *nxf3* mutants, 10 out of 60 germline transposons were derepressed more than fourfold (Fig. 3A, bottom), and these defects in transposon silencing were comparable with those observed in *rhi* and *moon* mutants (Supplemental Fig. S2C, left and middle). Notably, *bootlegger* and *nxf3* mutants had very similar impacts overall, considering effects on genes and transposons (Supplemental Fig. S2C, right).

Previous reports showed that loss of the RDC complex results in down-regulation of piRNA precursors derived from dual-strand clusters (Mohn et al. 2014; Zhang et al. 2014). To evaluate the potential effects of *bootlegger* and *nxf3* mutations on piRNA cluster levels, we extracted reads uniquely mapping to piRNA source loci and binned them into 1-kb windows (see the Materials and Methods for details). Loss of either Bootlegger or Nxf3 had a pronounced impact on the levels of piRNA precursors derived from dual-strand clusters, though the effects were milder than those observed in *rhi* mutants (Fig. 3B). Interestingly, the levels of piRNA precursors from cluster *42AB* were reduced more severely in *bootlegger* mutant ovaries then they were in *nxf3* knockouts (Fig. 3B,C). These data are in line with Bootlegger acting upstream of Nxf3 and suggest a function of Bootlegger in cluster transcription or transcript stability in addition to its role in recruitment of Nxf3. In contrast, the reduction of RNA from piRNA cluster *80F* was comparable between *bootlegger* and *nxf3* mutants (Supplemental Fig. S2D). Of note, the levels of transcripts derived from the unistrand clusters *flam* and *20A* remained unchanged (Fig. 3B; Supplemental Fig. S2D).

We sequenced small RNA libraries prepared from total RNA from ovaries of *bootlegger* and *nxf3* mutant flies and observed a global reduction in repeat-derived piRNAs in both mutants (Supplemental Fig. S3A). We specifically extracted reads mapping to piRNA source loci using the same binning strategy described above. Levels of piRNAs mapping to the major dual-strand clusters were severely reduced in *bootlegger* and *nxf3* mutants (Fig. 3D,E; Supplemental Fig. S3B). Compared with wild-type controls, we observed a strong reduction of sense and antisense piRNAs from cluster *42AB* upon loss of either Bootlegger or Nxf3, similar to *moon* mutants but weaker than *rhi* mutants (Fig. 3D,E). In contrast, piRNAs mapping to clusters *20A* and *flam* remained unchanged, as were gene-derived piRNAs (Fig. 3D; Supplemental Fig. S3B). Considered together, our data are consistent with a model in which Nxf3 and Bootlegger are specifically required for the production of piRNAs from dual-strand cluster transcripts.

### Nxf3 promotes export of piRNA cluster transcripts to cytoplasmic processing sites

**Figure 4.**
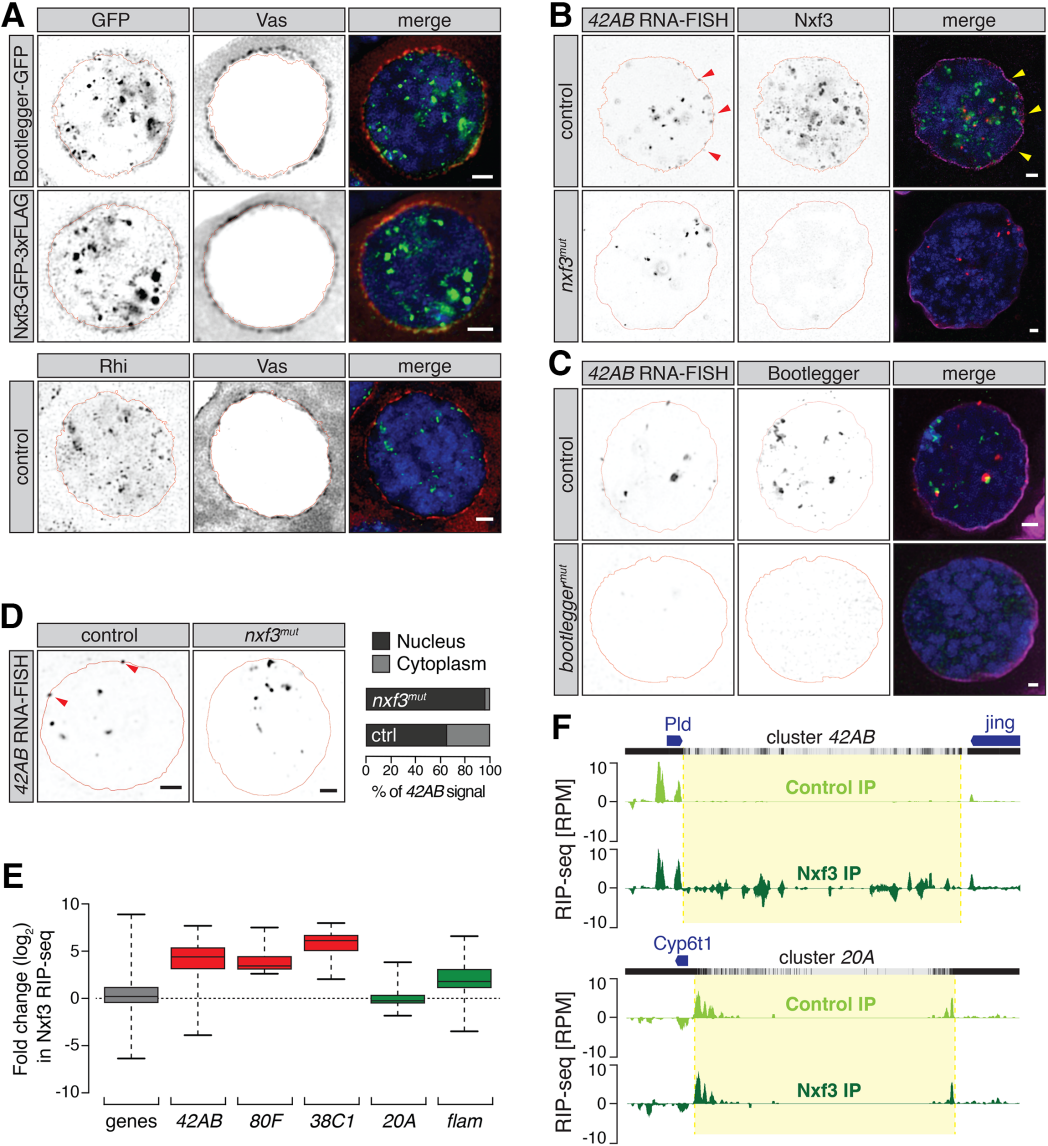
Nxf3 promotes dual-strand piRNA cluster export. (*A*) Expression and localization of Bootlegger-GFP (*top*), Nxf3-GFP-3xFlag (*middle*), and Rhi in nurse cell nuclei are shown by immunofluorescence and costained with Vas. (Green) GFP/Rhi; (red) Vas; (blue) DNA. Scale bar, 2 µm. (*B*) Expression and localization of Nxf3 protein and cluster *42AB* RNA are shown by immunofluorescence and RNA fluorescent in situ hybridization (FISH) in control and *nxf3*^*mut*^ nurse cell nuclei. (Green) Nxf3; (red) cluster *42AB* RNA; (magenta) Lamin, (blue) DNA. Scale bar, 2 µm. (*C*) As in *B* but showing expression and localization of Bootlegger protein and cluster *42AB* RNA in control and *bootlegger*^*mut*^ nurse cell nuclei. (Green) Bootlegger; (red) cluster *42AB* RNA; (magenta) Lamin, (blue) DNA. Scale bar, 2 µm. (*D*, *left*) Expression and localization of piRNA cluster *42AB* transcripts are shown by RNA-FISH in control and *nxf3*^*mut*^ nurse cell nuclei. Scale bar, 2 µm. (*Right*) The number of nuclear and cytoplasmic foci was quantified for control (*n* = 92) and *nxf3* mutant (*n* = 97) nurse cell nuclei. (*E*) Box plots showing changes in Nxf3 RIP-seq (RNA immunoprecipitation sequencing) levels compared with control for the indicated categories (1-kb bins for piRNA cluster transcripts *42AB*, *80F*, *38C1*, *20A*, and *flam*). (Gray) Protein-coding genes; (red) dual-strand clusters; (green) unistrand clusters. (*F*) UCSC genome browser shot displaying profiles of Nxf3 RIP-seq levels of reads uniquely mapping to cluster *42AB* (*top*) and cluster *20A* (*bottom*) in the indicated conditions. (Light green) Control immunoprecipitation; (dark green) Nxf3 immunoprecipitation. Reads per million (RPM) are shown. The mappability of 50-bp reads is shown *above*.

Unlike RDC components and Moon, Bootlegger and Nxf3 could be detected outside nuclei in addition to their presence in nuclear foci (Fig. 1D,E). We confirmed their perinuclear localization by costaining with Vas, which marks nuage in nurse cell nuclei (Lim and Kai 2007). While most Nxf3 foci were detected in nuclei, some signal overlapped with Vas staining (Fig. 4A; Supplemental Fig. S4A). The same pattern was observed for Bootlegger, whereas Rhi was present only in nuclear foci (Fig. 4A; Supplemental Fig. S4A), as reported previously (Klattenhoff et al. 2009; Mohn et al. 2014; Zhang et al. 2014). These results suggest that Bootlegger and Nxf3 transit between the nucleus and cytoplasm, where they are present in nuage, the sites of piRNA biogenesis in germ cells.

Given that Nxf3 is part of the nuclear export factor family, the localization pattern of Nxf3 protein in nuclei and nuage and the effects of *nxf3* loss on cluster transcripts and mature piRNAs suggested a role for Nxf3 in the export of piRNA precursors. To test this hypothesis, we performed RNA fluorescent in situ hybridization (FISH) for cluster *42AB* transcripts in wild-type control, *nxf3*^*mut*^, and *bootlegger*^*mut*^ ovaries. No signal was detected in nurse cells of *bootlegger* mutants, whereas *nxf3*^*mut*^ ovaries retained only staining of nuclear foci (Fig. 4B,C; Supplemental Fig. S4B). In control ovaries, the majority of the signal detected corresponded to nuclear foci; however, we also detected cytoplasmic foci (typically one to two foci per stack), corresponding to exported precursors (Fig. 4B-D, left). While *nxf3* mutants did not affect the nuclear foci, we observed a substantial reduction of cluster *42AB* transcript signals outside of the nuclei (Fig. 4D). In control flies, 35% of cluster *42AB* RNA signal was cytoplasmic, compared with only 4% of signal in the cytoplasm in *nxf3* mutants, highlighting a critical function of Nxf3 in piRNA precursor export (Fig. 4D, right).

Given the implication of Nxf3 function in dual-strand cluster export, we tested whether there was a direct interaction between Nxf3 and piRNA cluster transcripts. We immunoprecipitated Nxf3 protein from control and *nxf3*^*mut*^ ovaries and analyzed the associated RNA by RIP-seq (RNA immunoprecipitation sequencing). We detected a strong enrichment for RNAs derived from dual-strand clusters in Nxf3 RIP, whereas transcripts of protein-coding genes were not enriched in comparison with the control (Fig. 4E). Transcripts from cluster *42AB* and cluster *80F* were enriched ∼20-fold, while the levels of the unistrand cluster *20A* were comparable between control and mutant ovaries (Fig. 4E,F; Supplemental Fig. S4C). These data indicate that Nxf3 binds predominantly to piRNA precursor transcripts derived from dual-strand clusters in nurse cells.

### Nxf3-mediated piRNA precursor export requires Nxt1, Bootlegger, and Crm1

**Figure 5.**
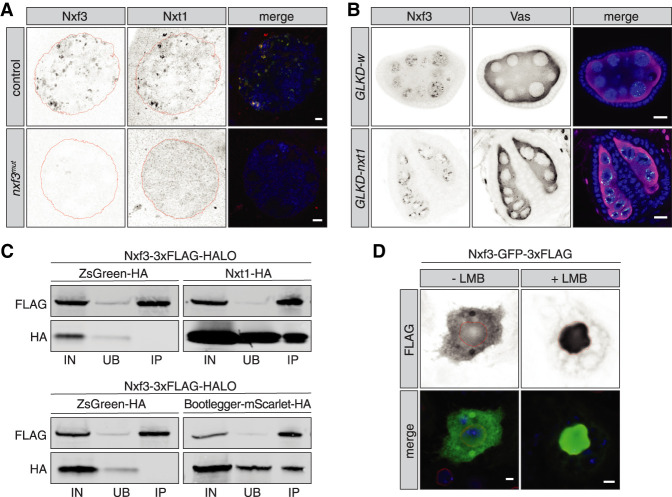
Nxf3 interacts with Nxt1 at piRNA cluster loci, and piRNA precursor export requires Crm1. (*A*) Expression and localization of Nxf3 and Nxt1 in control and *nxf3*^*mut*^ nurse cell nuclei is shown by immunofluorescence. (Green) Nxf3; (red) Nxt1; (blue) DNA. Scale bar, 2 µm. (*B*) Expression and localization of Nxf3 and Vas in egg chambers upon germline-specific knockdown (GLKD) of *nxt1* or *w* (control) are shown by immunofluorescence. (Green) Nxf3; (magenta) Vas; (blue) DNA. Scale bar, 10 µm. (*C*) Western blot analyses of Flag tag pull-down from lysates of S2 cells transfected with the indicated expression constructs. (IN) Input (10%); (UB) unbound (2.5%); (IP) immunoprecipitate (50%). (*D*) Expression and localization of the Nxf3-GFP-3xFlag construct in S2 cells treated with leptomycin B (LMB) or control treatment are shown by immunofluorescence. (Green) Flag; (red) Lamin; (blue) DNA. Scale bar, 2 µm.

Eukaryotic cells use several strategies to ensure proper mRNA export (Björk and Wieslander 2017). Canonical mRNA export by Nxf1 depends on heterodimer formation with its cofactor, Nxt1, and this interaction is mediated through their NTF2-like domains (Herold et al. 2001; Köhler and Hurt 2007; Stewart 2010). Nxf3 is also predicted to contain a NTF2-like domain (Fig. 1B); thus, we probed for a potential interaction between Nxf3 and Nxt1. In wild-type ovaries, Nxf3 and Nxt1 colocalized to distinct foci of nurse cell nuclei (Fig. 5A), while Nxt1 showed additional, dispersed signal throughout the nucleus. In *nxf3*^*mut*^ ovaries, all nuclear Nxt1 foci were lost, while the dispersed signal remained unchanged (Fig. 5A). Next, we analyzed the localization of Nxf3 upon *nxt1* germline knockdown. Depletion of Nxt1 leads to severe morphological defects of the ovaries, likely due to its function in general mRNA export (Fig. 5B). To identify the germ cells in Nxt1-depleted ovaries, we used Vas staining and found that Nxf3 was still detected in germ cell nuclei (Fig. 5B). Thus, our data are consistent with a model in which Nxf3 and Nxt1 likely form a complex in nurse cell nuclei, with Nxf3 recruiting Nxt1 to piRNA cluster transcripts. To test whether Nxf3 and Nxt1 interact, we expressed both proteins in Schneider 2 (S2) cells. Coimmunoprecipitation (co-IP) and Western blot analysis showed an association between Nxf3 and Nxt1 (Fig. 5C, top). Since Bootlegger recruitment to piRNA clusters depends on RDC (Fig. 2A) and since Nxf3 fails to associate with RDC foci in the absence of Bootlegger (Fig. 2B), we reasoned that Bootlegger could be responsible for bringing Nxf3 to piRNA precursors via an interaction between these proteins. Indeed, Nxf3 and Bootlegger coimmunoprecipitated in S2 cells, and reciprocal co-IP from ovaries confirmed that Bootlegger and Nxf3 interact in vivo (Fig. 5C, bottom; Supplemental Fig. S4D), leading us to conclude that Bootlegger recruits Nxf3—which subsequently brings Nxt1—to dual-strand clusters, ultimately linking RDC-dependent cluster transcription with Nxf3-dependent transcript export.

Nxf1 is responsible for canonical mRNA export; however, the absence of key mRNA processing such as splicing and polyadenylation led us to investigate alternative export mechanisms for piRNA precursor transcripts. The nuclear exportin Crm1 is largely responsible for the export of proteins but has also been reported to assist in nuclear export of RNAs via some nuclear export factor family proteins, including human Nxf3 (Yang et al. 2001; Köhler and Hurt 2007). Moreover, both mouse and *Xenopus* Nxt1 were shown to directly bind Ran-GTP and activate Crm1-dependent nuclear export (Ossareh-Nazari et al. 2000; Black et al. 2001), leading us to postulate that Crm1 might facilitate Nxf3–Nxt1-dependent export of piRNA precursors from *Drosophila* nurse cell nuclei. To test this hypothesis, we exploited a small molecule drug, leptomycin B (LMB), which specifically blocks Crm1-dependent export (Kudo et al. 1999). We transfected S2 cells with a Nxf3-GFP-3xFlag expression construct and treated the cells with LMB for 12 h. In the absence of LMB, the Nxf3 fusion protein was uniformly distributed in nuclei and the cytoplasm (Fig. 5D). In contrast, treatment with LMB resulted in accumulation of Nxf3-GFP-3xFlag in nuclei (Fig. 5D). These results suggest that Nxf3 contains an intrinsic nuclear localization signal but can be shuttled to the cytoplasm. Considered together, our data indicate that Nxf3-mediated export of piRNA precursors depends on Crm1.

## Discussion

The transcription and export of piRNA precursor transcripts require a highly specialized machinery that must assemble correctly at dual-strand cluster loci, initiate noncanonical transcription, and license and transport these transcripts (which lack features of processed and export-competent mRNAs) to the cytoplasm, where they are processed into mature piRNAs. How each step is achieved and how the elements involved interact are yet to be fully understood.

**Figure 6.**
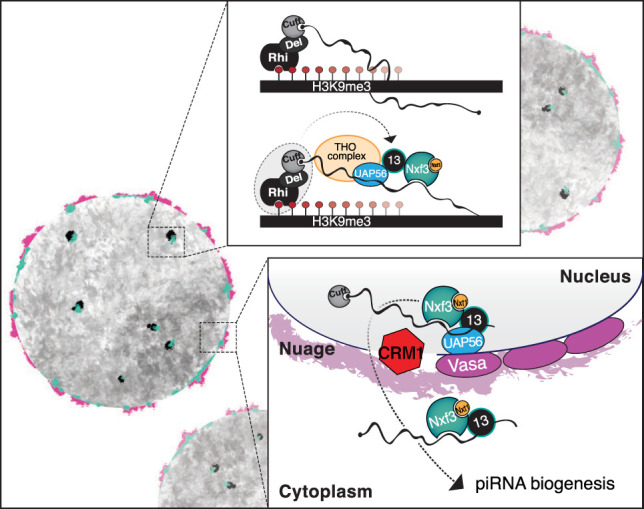
Model of piRNA cluster transcript generation and export. In germ cell nuclei of the *Drosophila* ovary, the RDC complex licenses dual-strand piRNA cluster transcription. Bootlegger is recruited to cluster loci via RDC components. (*Top inset*) Bootlegger instruments the recruitment of UAP56 followed by the THO complex and Nxf3. Nxf3 associates with piRNA precursors and Nxt1. Subsequently, Bootlegger, Nxt1, and Nxf3 in complex with piRNA precursors transit to nuage in a Crm1-dependant process. (*Bottom inset*) Following their export, dual-strand piRNA cluster transcripts are funneled into the piRNA biogenesis machinery.

Here we show that export of piRNA precursors from dual-strand clusters in nurse cells depends on a specific mechanism that requires Bootlegger, a protein without known domains, and the nuclear export factor Nxf3 (Fig. 6). We found that Bootlegger is important for either the synthesis or stability of transcripts from dual-strand piRNA clusters and is required for Nxf3 recruitment to dual-strand piRNA cluster loci. Analysis of RNA-seq, small RNA-seq, and RIP-seq for Nxf3, combined with immunofluorescence and RNA-FISH analyses, provides evidence that Nxf3 binds to and transports piRNA precursor transcripts from their sites of transcription (piRNA clusters) to the sites where piRNA processing takes place (nuage). We further found that Nxf3-mediated export depends on Crm1. These findings are all in agreement with those of ElMaghraby et al. (2019), who also identified Nxf3 and Bootlegger as critical facilitators of piRNA precursor export.

How Bootlegger is recruited to piRNA cluster loci remains to be determined. One plausible mechanism is via a direct protein–protein interaction with one or more components of the RDC complex, which specifies piRNA clusters upon recognition of H3K9me3 marks by Rhi. In support of this hypothesis, an interaction between Bootlegger and Del was observed by a yeast two-hybrid screen (ElMaghraby et al. 2019); however, whether this complex forms in ovaries requires further examination. Recruitment of Nxf3 in turn requires Bootlegger, likely also through direct protein–protein interaction. It will be important to uncover the precise contacts that drive this recruitment and probe whether RNA binding by Nxf3 (and/or Bootlegger) might contribute to complex formation. Nxf3 likely binds cluster RNAs through its cargo-binding domain, which is composed of an RNA-binding domain and LRRs. However, how specific binding of piRNA precursors is achieved remains a mystery. In fact, how exclusivity for the three nuclear export factor proteins with annotated functions (Nxf1–3) is achieved is a critical question for the future.

Eukaryotic cells use various different RNA export machineries, depending on the class of cargo RNA. Canonical mRNAs, which carry 5′ caps, have undergone splicing, and carry 3′ poly(A) tails, are exported via the Nxf1–Nxt1 pathway. Another extreme example are pre-microRNAs (pre-miRNAs), which are specifically recognized via their secondary structure and exported by Exportin-5 (Yi et al. 2003). A subset of RNAs is exported via a Crm1-dependant mechanism. Although Crm1 is typically involved in protein export, it can function in RNA export via adapter proteins. For example, Crm1 binds to the Cap-binding complex and the adapter protein PHAX to export ∼200-nt sized small nuclear RNAs (Ohno et al. 2000). Here we show that Nxf3-mediated export of piRNA precursor transcripts also requires Crm1. The role of Nxt1 in piRNA precursor export is not yet clear. It is plausible that Nxt1 is required for export by Nxf3 or might function in cotranscriptional recruitment to cargo RNAs. In this regard, it is striking that Nxt1 and also UAP56 and THO complex components are concentrated at piRNA clusters (Zhang et al. 2012, 2018) even though these proteins are reportedly essential for general mRNA export.

The work presented here and other emerging realizations (Batki et al. 2019; ElMaghraby et al. 2019; Fabry et al. 2019; Murano et al. 2019; Zhao et al. 2019) begin to paint a picture in which the evolutionary pressures exerted by the need to control mobile genetic elements have resulted in exaptation and dedication of nuclear export factor family members to the piRNA pathway. This specialization has taken different forms. The most easily understood is the adaptation of Nxf3 to export a specific class of noncanonically transcribed RNAs (ElMaghraby et al. 2019), whereas the precise mechanism by which Nxf2 acts as a cotranscriptional silencing factor remains more mysterious (Batki et al. 2019; Fabry et al. 2019; Murano et al. 2019; Zhao et al. 2019). Ultimately, an understanding of how these proteins have assumed new roles through evolution will require a detailed understanding of how they are recruited to particular transcripts and how they mediate their downstream effects.

## Materials and methods

### Fly stocks and handling

All flies were kept at 25°C on standard cornmeal or propionic food. CG13741-GFP, extended Nxf3-GFP-3xFlag, annotated Nxf3-GFP-3xFlag, *CG13741* mutant alleles (*CG13741^Δ1^* and *CG13741^Δ2^*), and the *nxf3* mutant allele (*nxf3*^*mut*^) were generated for this study (see below). Control *w*^*1118*^ flies were a gift from the University of Cambridge Department of Genetics Fly Facility. For germline-specific knockdowns, we used a stock containing a UAS::Dcr2 transgene and a nos::GAL4 driver (Czech et al. 2013) and shRNA and dsRNA lines from the Bloomington *Drosophila* Stock Center and Vienna *Drosophila* Resource Center. Fertility of mutant females was scored by crossing 10 freshly hatched females to five *w*^*1118*^ males and counting the number of eggs laid in 12-h periods and pupae that developed after 7 d. Details on all fly stocks used in this study are listed in Supplemental Table S1.

### Generation of mutant and transgenic fly strains

Frameshift mutant alleles of *CG13741* and *nxf3* were generated by injecting pCFD4 (a gift from Simon Bullock; Addgene plasmid 49411; http://n2t.net/addgene:49411; RRID Addgene_49411) (Port et al. 2014) containing two gRNAs against *CG13741* and *nxf3* into embryos expressing vas::Cas9 (Bloomington stock, 51323). CG13741-GFP was cloned in an in-house-generated transgenesis vector for ΦC31-mediated integration, expressed under the *Drosophila melanogaster ubiquitin* promoter (pUBI), and integrated into *attP40* sites on chromosome 2 (stock 13–20). Extended Nxf3-GFP-3xFlag and annotated Nxf3-GFP-3xFlag were cloned in an in-house-generated transgenesis vector for ΦC31-mediated integration, expressed under the *D. melanogaster act5c* promoter (pAct5c), and integrated into *attP40* sites on chromosome 3 (stock 13–18). Microinjection and fly stock generation were carried out by the University of Cambridge Department of Genetics Fly Facility. Mutant flies were identified by genotyping PCRs and confirmed by Sanger sequencing.

### RNA isolation and qPCR analysis

Samples were lysed in 1 mL of Trizol, and RNA was extracted according to the manufacturer's instructions. One microgram of total RNA was treated with DNase I (Thermo Fisher Scientific) and reverse-transcribed with the SuperScript III first strand synthesis kit (Thermo Fisher Scientific) using oligo(dT)_20_ primers. Real-time PCR (qPCR) experiments were performed with a QuantStudio real-time PCR Light Cycler (Thermo Fisher Scientific). Transposon levels were quantified using the ΔΔCT method (Livak and Schmittgen 2001) and normalized to *rp49*, and fold changes were calculated relative to the indicated controls. All oligonucleotide sequences are in Supplemental Table S2.

### Small RNA-seq library preparation

Small RNA libraries were generated as described previously with slight modifications (McGinn and Czech 2014). Briefly, 18- to 29-nt sized small RNAs were purified by PAGE from 12 µg of total RNA from ovaries. Next, the 3′ adapter (containing four random nucleotides at the 5′ end) (Jayaprakash et al. 2011) was ligated using T4 RNA ligase 2 and truncated KQ (New England Biolabs). Following recovery of the products by PAGE purification, the 5′ adapter (containing four random nucleotides at the 3′ end) was ligated to the small RNAs using T4 RNA ligase (Ambion). Small RNAs containing both adapters were recovered by PAGE purification, reverse-transcribed, and PCR-amplified. Libraries were sequenced on an Illumina HiSeq 4000 (Illumina).

### RNA-seq library preparation

Five micrograms of RNA was cleaned up using RNeasy miniprep column (Qiagen) according to the manufacturer's instructions. One microgram of input RNA was used for ribosomal RNA (rRNA) removal with Ribo-Zero rRNA removal kit (human/mouse/rat) (Illumina). Libraries were generated with the NEBNext Ultra Directional RNA library preparation kit for Illumina (New England Biolabs) according to the manufacturer's instructions. The pooled libraries were quantified with KAPA library quantification kit for Illumina (Kapa Biosystems) and sequenced on an Illumina HiSeq 4000 (Illumina).

### RIP-seq library preparation

RIP-seq was adapted from Hendrickson et al. (2016). Ovaries from ∼100 *w*^*1118*^ or *nxf3*^*mut*^ flies (3–5 d old) were dissected in ice-cold PBS and fixed with 0.1% PFA for 20 min followed by quenching with equal volumes of 125 mM glycine. Fixed ovaries were lysed in 1 mL of RIPA buffer (supplemented with complete protease inhibitors [Roche] and 40 U/mL RNasin Plus [Promega]) and homogenized using a motorized pestle. Lysates were incubated for 20 min at 4°C on a tube rotator and sonicated with a Bioruptor Pico (three cycles of 30 sec on/30 sec off). After lysis, lysates were spun at maximum speed for 10 min at 4°C. Lysates were diluted by adding equal volumes of RIP binding/wash buffer (150 mM KCl, 25 mM Tris at pH 7.5, 5 mM EDTA, 0.5% NP-40, 0.5 mM DTT, supplemented with protease inhibitors, RNasin Plus 1:1000). Lysates were precleared using 40 µL of Pierce protein A/G beads for 1 h at 4°C, and Nxf3 proteins were immunoprecipitated by incubation with 40 µL of Nxf3 antibody overnight at 4°C. Eighty microliters of Pierce A/G magnetic beads was then added to the lysates and incubated for 3 h at 4°C. An aliquot of precleared input lysate was saved for RNA isolation and library preparation. Following three washes in RIP binding/wash buffer, immunoprecipitation and input samples were reverse-cross-linked in 1× reverse cross-linking buffer (PBS, 2% N-lauroyl sarcosine, 10 mM EDTA, 5 mM DTT) and proteinase K. RNA isolation was performed using Trizol, and 50 ng of input or immunoprecipitation RNA was used for rRNA removal using RiboGone mammalian (Clontech) and library preparation using the SMARTer stranded RNA-seq kit (Clontech). DNA libraries were quantified with KAPA library quantification kit for Illumina (Kapa Biosystems) and deep-sequenced with Illumina HiSeq 4000 (Illumina).

### Ovary immunostaining

Fly ovaries were dissected in ice-cold PBS, fixed in 4% PFA for 14 min at room temperature, and permeabilized with three 10-min washes in PBS with 0.3% Triton (PBS-Tr). Samples were blocked in PBS-Tr with 1% BSA for 2 h at room temperature and incubated overnight at 4°C with primary antibodies in PBS-Tr and 1% BSA. After three 10-min washes at room temperature in PBS-Tr, secondary antibodies were incubated overnight at 4°C in PBS-Tr and 1% BSA. After four 10-min washes in PBS-Tr at room temperature (DAPI was added during the third wash) and two 5-min washes in PBS, samples were mounted with ProLong Diamond antifade mountant (Thermo Fisher Scientific, P36961) and imaged on a Leica SP8 confocal microscope. Images were deconvoluted using Huygens Professional. The following antibodies were used: anti-GFP (ab13970), anti-Nxf3, anti-CG13741/Bootlegger, anti-Piwi (Brennecke et al. 2007), anti-Aub (Senti et al. 2015), anti-Ago3 (Senti et al. 2015), anti-Vas (Developmental Studies Hybridoma Bank [DSHB], anti-vasa), anti-Lamin (DSHB, ADL67.10), anti-UAP56 (Eberl et al. 1997), and anti-Nxt1 (Herold et al. 2001).

### Ovary immunostaining/RNA-FISH

Fly ovaries were dissected in ice-cold PBS, fixed in 4% PFA for 14 min at room temperature, and permeabilized with three 10-min washes in PBS-Tr. Samples were blocked in PBS-Tr with 1% BSA (supplemented with RNasin Plus [Promega] 1:1000) for 2 h at room temperature and incubated overnight at 4°C with primary antibodies in PBS-Tr and 1% BSA (supplemented with RNasin 1:1000). After three 10-min washes at room temperature in PBS-Tr, secondary antibodies were incubated overnight at 4°C in PBS-Tr and 1% BSA (supplemented with RNasin Plus [Promega] 1:1000). After four 10-min washes in PBS-Tr, ovaries were fixed in fixation solution (4% PFA, 0.15% Triton in PBS) for 20 min. Ovaries were washed three times for 10 min in PBS-Tr and permeabilized overnight in 70% ethanol. Ovaries were rehydrated in 2× SSC for 5 min. For cluster *42AB* RNA-FISH, samples were prehybridized in 500 µL of 30% hybridization buffer (Molecular Instruments) for 30 min at 37°C. Ovaries were resuspended in 30% hybridization buffer (Molecular Instruments) containing 1 µL of 2 µM each probe set (even and odd) and incubated for 24 h at 37°C. Samples were washed four times for 15 min with 500 µL of 30% probe wash buffer (Molecular Instruments) followed by three 5-min washes with 5× SSCT (0.1% Tween-20 in 5× SSC) at room temperature. Samples were preamplified in 500 µL of amplification buffer (Molecular Instruments) for 30 min at room temperature. Thirty picomoles of fluorescently labeled hairpin was heated for 90 sec at 95°C and cooled for 30 min to room temperature. Amplification solution was prepared by adding the fluorescently labeled hairpins to 500 µL of amplification buffer. Samples were incubated overnight at room temperature in 125 µL of amplification solution. Samples were washed twice for 5 min (DAPI was added to the second wash at 1:5000), twice for 30 min, and once for 5 min with 5× SSCT. Samples were mounted with ProLong Diamond antifade mountant (Thermo Fisher Scientific, P36961) and imaged on a Leica SP8 confocal microscope. Images were deconvoluted using Huygens Professional. The following antibodies were used: anti-Nxf3, anti-CG13741, and anti-Lamin (DSHB, ADL67.10).

### Image analysis

Image analysis was carried out in Fiji. For the display of germ cell nuclei, the nuclear outline was derived from either DAPI or Lamin stain. Quantification of RNA-FISH was performed by counting nuclear and cytoplasmic foci with Fiji after size and intensity thresholding; the nuclear outline was derived from DAPI staining.

### Co-IP from ovaries

Ovaries from 50 Bootlegger-GFP and 50 control flies were dissected in ice-cold PBS, lysed in 200 µL of co-IP lysis buffer (20 mM Tris-HCl at pH 7.5, 150 mM NaCl, 2 mM MgCl_2_, 10% glycerol, 1 mM DTT, 0.1 mM PMSF, 0.2% NP-40 supplemented with complete protease inhibitors), and homogenized using a motorized pestle. Lysates were cleared at 16000*g* for 5 min, and the residual pellet was re-extracted with the same procedure. GFP-tagged proteins were immunoprecipitated by incubation with 25 µL of GFP-Trap magnetic agarose beads (Chromotek) overnight at 4°C on a tube rotator. Nxf3 proteins were immunoprecipitated by incubation with 10 µL of anti-Nxf3 antibody overnight at 4°C on a tube rotator. Ten microliters of Pierce A/G magnetic beads was the added to the lysates and incubated for 3 h at 4°C. The beads were washed six times with lysis buffer. Samples were eluted using 20 µL of 2× sample buffer (Invitrogen) by boiling beads for 3 min and analyzed by Western blot.

### Western blot

Protein concentration was measured using a Direct Detect infrared spectrometer (Merck). Twenty micrograms of proteins was separated on a NuPAGE 4%–12% Bis-Tris gel (Thermo Fisher Scientific). Proteins were transferred with an iBLot2 device (Invitrogen) on a nitrocellulose membrane and blocked for 1 h in 1× Licor TBS blocking buffer (Licor). Primary antibodies were incubated overnight at 4°C. Licor secondary antibodies were incubated for 45 min at room temperature, and images were acquired with an Odyssey CLx scanner (Licor) using secondary antibodies conjugated to infrared dyes from LiCor. The following primary antibodies were used: anti-CG13741, anti-Nxf3, anti-Tubulin (ab18251), anti-GFP (ab13790), anti-Flag (Sigma, F1804), and anti-HA (Cell Signaling, 3724S).

### Nxf3 pull-down and mass spectrometry

Ovaries from ∼100 control flies (*w*^*1118*^ strain; 3–5 d old) were dissected in ice-cold PBS, lysed in 400 µL of RIPA buffer (supplemented with complete protease inhibitors [Roche]), and homogenized using a motorized pestle. Lysates were incubated for 20 min at 4°C on a tube rotator and sonicated with a Bioruptor Pico (three cycles of 30 sec on/30 sec off). After lysis, lysates were spun at maximum speed for 10 min at 4°C. Lysates were precleared using 40 µL of Pierce protein A/G beads for 1 h at 4°C, and Nxf3 proteins were immunoprecipitated by incubation with 40 µL of Nxf3 antibody overnight at 4°C. Eighty microliters of Pierce A/G magnetic beads was then added to the lysates and incubated for 3 h at 4°C. Beads were washed three times for 10 min with wash buffer (150 mM KCl, 25 mM Tris at pH 7.5, 5 mM EDTA, 0.5% NP-40, 0.5 mM DTT supplemented with complete protease inhibitors). Beads were rinsed twice with 100 mM ammonium bicarbonate and submitted for mass spectrometry. Samples were analyzed on a Q-Exactive HF mass spectrometer (Thermo Fisher Scientific) after trypsin digestion.

Spectral .raw files were processed with the SequestHT search engine on Thermo Scientific Proteome Discoverer 2.2. Data were searched first against a custom FlyBase database (“dmel-all-translation-r6.24”) at 1% spectrum-level false discovery rate (FDR) criteria using Percolator (University of Washington). Data were also searched against a custom database including only the N-terminal extended version of Nxf3 (for the amino acid sequence, see Supplemental Table S3). MS1 mass tolerance was constrained to 20 ppm, and the fragment ion mass tolerance was set to 0.02 Da. Oxidation of methionine residues (+15.995 KDa) AND deamidation (+0.984) of asparagine and glutamine residues were included as dynamic modifications. The precursor ion quantifier node (Minora feature detector) included a minimum trace length of 5 and maximum ΔRT of isotope pattern 0.2 min. For calculation of precursor ion intensities, feature mapper was set true for RT alignment (mass tolerance of 10 ppm). Precursor abundance was quantified based on intensity, and the level of confidence for peptide identifications was estimated using the Percolator node with a strict FDR at *q*-value < 0.01.

### Cell culture

*Drosophila* S2 cells were purchased from Thermo Fisher Scientific and grown at 26°C in Schneider medium supplemented with 10% FBS. S2 cells were transfected using Effectene (Qiagen) according to the manufacturer's instructions. S2 cells were treated with LMB at final concentration of 40 ng/mL for 12 h.

### S2 cell immunostaining

Cells were plated on concanavalin A-coated coverslips, fixed for 15 min in 4% PFA, permeabilized for 10 min in PBS with 0.2% Triton (PBST), and blocked for 30 min in PBS, 0.1% Tween-20, and 1% BSA. Primary antibodies were diluted in PBS, 0.1% Tween-20, and 0.1% BSA and incubated overnight at 4°C. After three 5-min washes in PBST, secondary antibodies were incubated for 1 h at room temperature. After three 5-min washes in PBST, DAPI was incubated for 10 min at room temperature and washed twice, and the coverslips were mounted using ProLong Diamond antifade mountant (Thermo Fisher Scientific, P36961) and imaged on a Leica SP8 confocal microscope (100× oil objective). The following antibodies were used: anti-Lamin (DSHB, ADL67.10) and anti-Flag (Cell Signaling, 14793).

### Co-IP from cell lysates

S2 cells were transfected with 3xFlag- and HA-tagged constructs. Cells were harvested 48 h after transfection and lysed in 250 µL of co-IP lysis buffer (Pierce) supplemented with Complete protease inhibitors (Roche). Protein lysates were diluted to 1 mL with co-IP lysis buffer, and the 3xFlag-tagged bait was immunoprecipitated by incubation with 20 µL of anti-Flag M2 magnetic beads (Sigma, M8823) for 2 h at 4°C on a tube rotator. The beads were washed three times for 15 min with TBS supplemented with protease inhibitors. Beads were then resuspended in 2× NuPAGE LDS sample buffer (Thermo Fisher Scientific) without reducing agent and boiled for 3 min at 90°C to elute immunoprecipitated proteins. Immunoprecipitations, unbound fractions, and input fractions were diluted to 1× NuPAGE LDS sample buffer concentration, and reducing agent was added. Samples were boiled for 10 min at 70°C before proteins were separated by Western blot.

### RNA-seq and RIP-seq analysis

Raw Fastq files generated by Illumina sequencing were analyzed by a pipeline developed in-house. In short, the five first bases and the last base of each 50-bp read were removed using Fastx trimmer (http://hannonlab.cshl.edu/fastx_toolkit). Following removal of rRNA mapped reads, high-quality reads were aligned to transposon consensus sequences followed by alignment to the *D. melanogaster* genome release 6 (dm6; downloaded from FlyBase) using STAR (Dobin et al. 2013). Genome multimapping reads were assigned randomly to one location using option “–outFilterMultimapNmax 1000 –outMultimapperOrder Random,” and nonmapping reads were removed. For genome-wide analyses, unique mapper reads were extracted to ensure unique locations of reads. Normalization was achieved by calculating RPM (reads per million). Reads mapping to genes were counted with HTSeq (Anders et al. 2015), and transposon-derived reads were calculated using a custom script. Differential expression analysis was performed using custom-built R scripts. piRNA clusters were divided into 1-kb bins, and unique reads mapping to these bins were counted using HTSeq. A pseudocount of 0.01 or 0.1 for RNA-seq and RIP-seq experiments, respectively, was then added to each bin before calculating log_2_ fold changes per bin.

### Small RNA-seq analysis

For small RNA-seq, adapters were clipped from raw Fastq files with Fastx_clipper (adapter sequence AGATCGGAAGAGCACACGTCTGAACTCCAGTCA) keeping only reads at least 23 bp in length. The first and last four bases were then trimmed using seqtk (https://github.com/lh3/seqtk). After removal of cloning markers and 2S rRNA mapped reads, alignment was performed as described above and normalized to miRNA reads in the control library (set to RPM). Only high-quality small RNA reads with a length between 18 and 30 bp were used for further analysis of small RNA profiles. piRNA distribution was calculated and plotted in R. piRNA clusters were divided into 1-kb bins, and unique reads mapping to these bins were counted using HTSeq. A pseudocount of 1 was added to each bin before calculating log_2_ fold changes per bin. Small RNA size profiles were plotted in R, and only unique mappers were used for small RNA distribution of piRNA clusters.

### Data availability

Sequencing data reported here have been deposited in Gene Expression Omnibus (GSE133528). Mass spectrometry data have been deposited to the PRIDE (Proteomics Identifications) archive (PXD014472).
